# Impact Evaluation of Malaria Control Interventions on Morbidity and All-Cause Child Mortality in Rwanda, 2000–2010

**DOI:** 10.4269/ajtmh.17-0281

**Published:** 2017-09-27

**Authors:** Erin Eckert, Lia S. Florey, Jon Eric Tongren, S. René Salgado, Alphonse Rukundo, Jean Pierre Habimana, Emmanuel Hakizimana, Kaendi Munguti, Noella Umulisa, Monique Mulindahabi, Corine Karema

**Affiliations:** 1 President’s Malaria Initiative (PMI), U.S. Agency for International Development (USAID), Washington, District of Columbia;; 2 ICF, Rockville, Maryland;; 3 Centers for Disease Control and Prevention (CDC), PMI, Accra, Ghana;; 4 Malaria and Other Parasitic Diseases Division (MOPDD), Rwanda Biomedical Center, Kigali, Rwanda;; 5 President’s Malaria Initiative (PMI), U.S. Agency for International Development (USAID), Kigali, Rwanda;; 6 Maternal and Child Survival Program (MCSP), Jhpiego, Kigali, Rwanda;; 7 Swiss Tropical and Public Health Institute and University of Basel, Switzerland

## Abstract

The impressive decline in child mortality that occurred in Rwanda from 1996–2000 to 2006–2010 coincided with a period of rapid increase of malaria control interventions such as indoor residual spraying (IRS); insecticide-treated net (ITN) distribution and use, and improved malaria case management. The impact of these interventions was examined through ecological correlation analysis, and robust decomposition analysis of contextual factors on all-cause child mortality. Child mortality fell 61% during the evaluation period and prevalence of severe anemia in children 6–23 months declined 71% between 2005 and 2010. These changes in malaria morbidity and mortality occurred concurrently with a substantial increase in vector control activities. ITN use increased among children under five, from 4% to 70%. The IRS program began in 2007 and covered 1.3 million people in the highest burden districts by 2010. At the same time, diagnosis and treatment with an effective antimalarial expanded nationally, and included making services available to children under the age of 5 at the community level. The percentage of children under 5 who sought care for a fever increased from 26% in 2000 to 48% in 2010. Multivariable models of the change in child mortality between 2000 and 2010 using nationally representative data reveal the importance of increasing ITN ownership in explaining the observed mortality declines. Taken as a whole, the evidence supports the conclusion that malaria control interventions contributed to the observed decline in child mortality in Rwanda from 2000 to 2010, even in a context of improving socioeconomic, maternal, and child health conditions.

## INTRODUCTION

**Figure 1.**
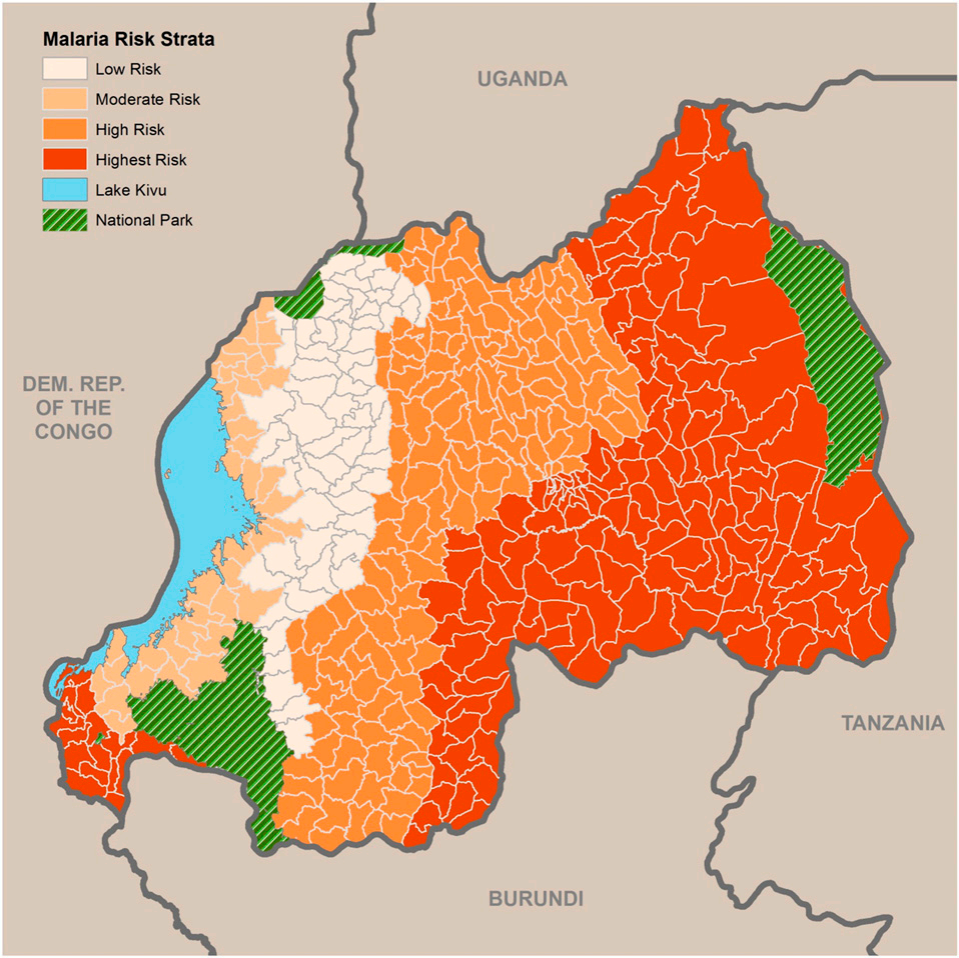
Malaria risk strata in Rwanda. Source: Malaria and Other Parasitic Diseases Division 2013.

Rwanda is a small (26,338 km^2^), land-locked country in the Great Lakes region of eastern Africa, bordered by Uganda, Burundi, the Democratic Republic of the Congo, and Tanzania. It has a population of approximately 11.8 million, making it the most densely populated country in continental Africa.^1^ The entire population is at risk for malaria, including an estimated 2.2 million children less than 5 years of age and 443,000 pregnant women per year. Rwanda is divided into four malaria ecologic zones based on altitude, climate, level of transmission, and disease vector prevalence (Figure 1). Malaria is mesoendemic in the plains and epidemic prone in the high plateaus and hills. Malaria transmission occurs year round with two peaks (May–June, November–December) in the endemic zones. Other factors that influence malaria transmission include access to and use of health-care services, high population density, population movement (from areas of low to high transmission), irrigation schemes, and cross-border movements of people.

Between 2000 and 2010, malaria morbidity declined in Rwanda through increasing implementation of malaria control interventions.^2,3^ The Rwandan Malaria and Other Parasitic Diseases Division (MOPDD), set the ambitious goal of achieving preelimination status and near zero malaria deaths by 2018 in their Malaria Strategic Plan.^4^ The strategies used included the adoption of new first-line pharmaceutical treatments, increased access to diagnostics with mandatory confirmatory testing via microscopy or rapid diagnostic tests (RDTs), targeted subnational indoor residual spraying (IRS), and improved access to and utilization of insecticide-treated nets (ITNs). Intermittent preventative treatment (IPTp) of pregnant women was only implemented for a short time, between 2004 and 2008.^5^

**Figure 2.**
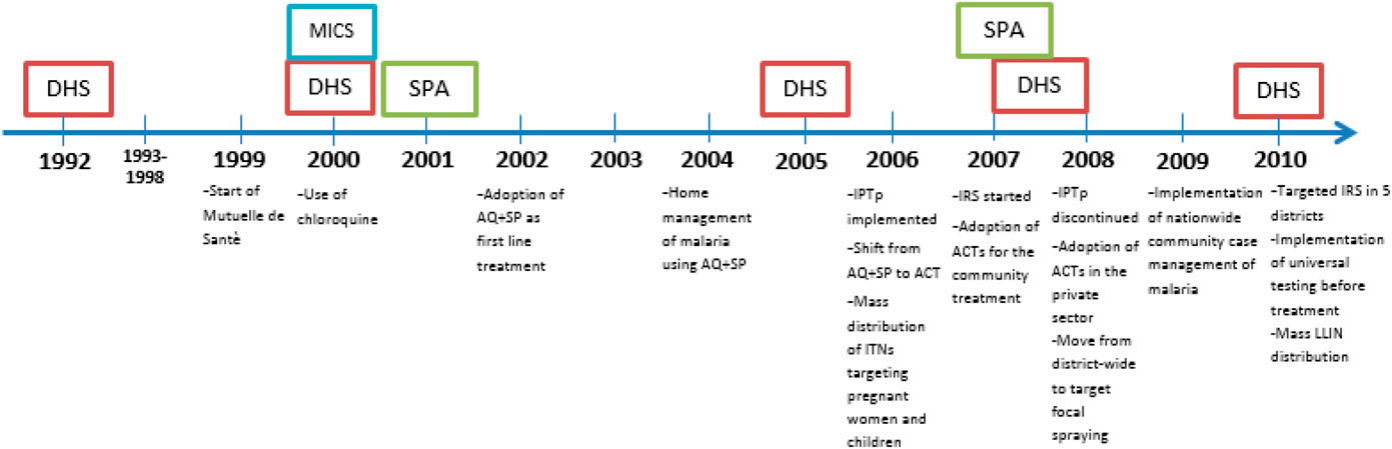
Timeline of data sources and malaria control interventions, 1992–2010. This figure appears in color at www.ajtmh.org.

Because of the serious disease burden caused by malaria and the extensive funding, both internal and external, which has been devoted to malaria control, there is a growing demand from policy-makers, program managers, donors, and researchers to measure the extent to which malaria control interventions have made an impact on malaria morbidity and child mortality. Earlier studies have documented the impact of multiple malaria control interventions on malaria incidence and prevalence. On Bioko Island, a statistically significant drop in parasite prevalence was observed following intensified vector control interventions (IRS, ITN).^6^ In Zanzibar, the use of effective ACTs, in combination with ITNs, resulted in significant reductions in malaria morbidity and mortality.^7^ In Tanzania, a comprehensive evaluation framework was applied to document the impact of a comprehensive malaria control program on malaria burden nationwide.^8,9^ This study uses nationally representative data to investigate the plausibility of a causal association between increasing implementation and utilization of malaria control interventions and declines in malaria-associated morbidity and all-cause child mortality (ACCM). Figure 2 shows a timeline with the dates of implementation of malaria control policies and interventions, and of relevant data sources. Similar methods of evaluating the impact of malaria control interventions have been applied in other countries in Africa, including several discussed in this supplement.

## MALARIA CONTROL POLICES AND IMPLEMENTATION

Vector control has long been one of the principal means of controlling malaria in Rwanda. In the 1990s, Rwanda began targeted distribution of bednets to pregnant women and children through antenatal clinics and vaccination campaigns. Long-lasting ITNs were introduced in 2006 and noninsecticidal nets banned in 2008.^10^

IRS began in Rwanda in the 1940s and continued through the 1960s. In 2007, Rwanda resumed implementation of IRS targeted to priority high-burden districts, using pyrethroid insecticides. In 2008, declining malaria burden led to a change in policy from district-wide coverage of IRS to targeted focal spraying (spraying only subsectors with the highest malaria burden instead of blanketing an entire district).^10^

To prevent malaria among pregnant women, a critical risk group in malaria endemic countries, Rwanda initially implemented a program of providing weekly preventive doses of chloroquine (CQ) to pregnant women in the 1990s. In 2004, following the World Health Organization recommendation for intermittent preventive treatment with sulfadoxine/pyrimethamine (IPTp-SP), Rwanda shifted to a policy of providing IPTp-SP to pregnant women. However, in 2008, a substantial decline in malaria transmission coupled with a high level of therapeutic failure of SP, led to the suspension of the IPTp program in favor of routine antenatal care (ANC), promotion of ITN use, and optimization of malaria case management among pregnant women.^11^

During the evaluation period, recommended first-line antimalarial treatment changed from CQ, to amodiaquine/sulfadoxine/pyrimethamine (AQ-SP) and finally, in 2006, to artemisinin combination therapies (ACTs) (specifically arthemether-lumefantrine). To address the issue of low utilization of health services, Rwanda implemented a program of home-based management of fever (HBMF) in 2004 and gradually expanded the program to 18 of 30 districts through 2007. In 2006, ACT was integrated into the HBMF program. In 2009, Rwanda implemented a policy of universal parasitological diagnosis by microscopy for patients with febrile illness in health centers and by RDT at the community level. According to the MOPDD, by 2010, all community health workers were using RDTs and 94% of all suspected cases (community and facility based) of malaria were tested.^2^

## METHODS

### Evaluation period.

The evaluation focused on the 2000–2010 period, during which most malaria control interventions were introduced and more than $379 million in donor funding allocated to Rwanda for malaria control. Before 2000, malaria control interventions were not widely available. As with much of Africa, malaria control was based primarily on untreated bednets and drug therapies, such as CQ and SP, which were rapidly losing effectiveness due to the development of resistance. Starting in 2000, the availability of more effective vector control interventions such as ITNs and IRS and improved case management based on diagnosis and effective treatment, rapidly increased across the country. This period also saw increased investment in malaria control from the national government, as well as international donors. Thus, the period 2000–2010 is a critical time in which to measure the impact of malaria control interventions on the burden of malaria in Rwanda.

### Evaluation design.

This evaluation is based on a before-and-after assessment, which uses a plausibility evaluation design to measure changes in malaria intervention coverage, malaria-related morbidity, and ACCM while considering other contextual determinants of child survival in Rwanda during 2000–2010.^12,13^ The choice of this evaluation design is discussed in detail by Ye and others in this supplement.

### Evaluation indicators.

The evaluation relied primarily on population-based indicators recommended by Roll Back Malaria Monitoring and Evaluation Reference Group, which include the following three primary impact measures: ACCM in children less than 5 years of age, malaria parasitemia among children 6–59 months of age, and severe anemia (Hb < 8 g/dL) prevalence in the same age group (Hb < 8 g/dL has been shown to be strongly correlated with malaria infection.^14^ ACCM is used as an impact indicator instead of malaria-specific mortality because malaria is a major contributor to child mortality in endemic countries, and using ACCM captures both the direct and indirect effects of malaria on mortality. Reliable and valid direct measures of malaria-specific mortality are not commonly available at the population level in most endemic countries.^13^ In addition, facility-based data are used to investigate trends in reported malaria cases, malaria incidence, and test positivity rates (TPRs), and published studies on the relationship between malaria control interventions and their impact are referenced where appropriate.

### Data sources.

Demographic and Health Surveys (DHS) conducted during 2000, 2005, 2007–2008, and 2010 were data sources for mortality in children under 5 years of age, malaria control intervention coverage indicators and some contextual factors.^15–18^ Other data sources included published documents containing data summaries from Rwanda’s Health Management Information System (HMIS), Community Information System (SIS-com), and Rwanda’s Integrated Disease Surveillance Reporting (IDSR) databases. Data on climate are drawn from the national meteorological archive.^19^ Subnational studies (e.g., MOPDD sentinel sites epidemiologic and entomologic surveys), therapeutic efficacy studies, and cross sectional and longitudinal studies from publications were also referenced.

### Plausibility assessment.

Changes in coverage with malaria control intervention and morbidity and mortality rates were calculated and summarized using the available nationally representative surveys and routine data cited earlier. Where survey data were used, standard errors were calculated for each estimate of intervention coverage or outcome measure to assess significance of changes between the baseline and endline of the evaluation. The direction and significance of changes in intervention coverage compared with the direction and significance of changes in malaria morbidity and in ACCM were compared with assess the plausibility of a causal relationship between the two. The likelihood of causality between the interventions and malaria morbidity and ACCM outcomes was considered increased in the follow instances:

1) If the magnitude of impact is consistent with established intervention efficacy, and geographic patterns of increased coverage (e.g., greater declines in mortality in areas where intervention intensity has increased).
2) If changes in the age pattern of intervention coverage are consistent with the age distribution of malaria-mediated morbidity and mortality.
3) If the timing of intervention scale-up aligns appropriately with a change in trends of impact measures.
4) If contextual factors influencing ACCM did not significantly impact the outcome during the evaluation period.

Where data permit, each of these analytical approaches was used in the evaluation. Changes in other determinants of child mortality (contextual factors) were also examined to assess their additional potential for effect on ACCM.

### Decomposition analysis.

To further explore which malaria control interventions and contextual factors contributed to the declines in ACCM seen across the evaluation period, a decomposition analysis was conducted. These models deconstruct the relative importance of changes in the distribution of variables between surveys and changes in the strength of effects of variables across two groups from the multivariate log probability models.^17,20^ The groups, in this case are surveys; the 2000 DHS and the 2010 DHS. In this analysis, the socioeconomic variables and measures of maternal and child health intervention coverage were analyzed for their proportional impact on all-cause child mortality. Multivariate log probability models were used following the mvdcmp procedure in Stata 13^22^ with a logit distribution.^23^

### Climate analysis.

Changes in rainfall and temperature were assessed as potential contextual factors impacting malaria control efforts in Rwanda. To analyze Rwanda’s climate in detail, high resolution data on monthly rainfall and average monthly temperature were obtained from satellite imagery and from the national meteorological archive, collectively known as the Enhanced National Climate Services database.^20^ These data were included in the Climate Suitability for Malaria Transmission model,^3^ designed to identify the number of months deemed suitable for malaria transmission. The model was then used to generate a time series Weighted Anomaly of Standardized Precipitation^24^ to examine whether the climate was more or less suitable for malaria transmission pre- and post-intervention.

## RESULTS

### Malaria control intervention coverage.

#### Insecticide-treated nets.

Before 2006, the national ITN distribution strategy targeted pregnant women and children under 5 years of age through ANC and the expanded program on immunization. In 2006, mass distribution campaigns for long-lasting insecticide treated nets (LLIN) were introduced and initially a nationwide measles vaccination campaign targeting children under 5 years of age was used to distribute 1.96 million LLINs. This was followed by the distribution of an additional 1.16 million LLINs in 2007 and 800,000 LLINs in 2009. In 2010, Rwanda’s national policy changed to universal coverage and a national campaign distributed almost five million LLINs. In total, over the evaluation period, 9.6 million nets were distributed in Rwanda.

**Figure 3.**
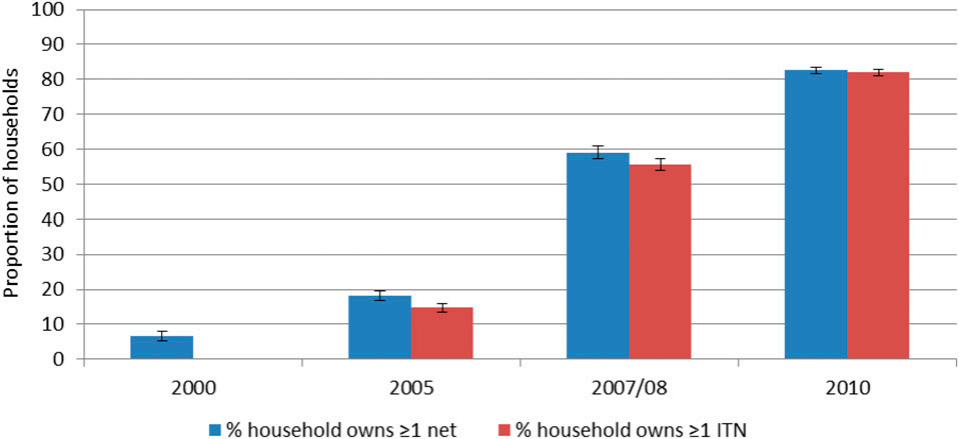
Household ownership of insecticide-treated nets, 2000–2010. This figure appears in color at www.ajtmh.org.

**Figure 4.**
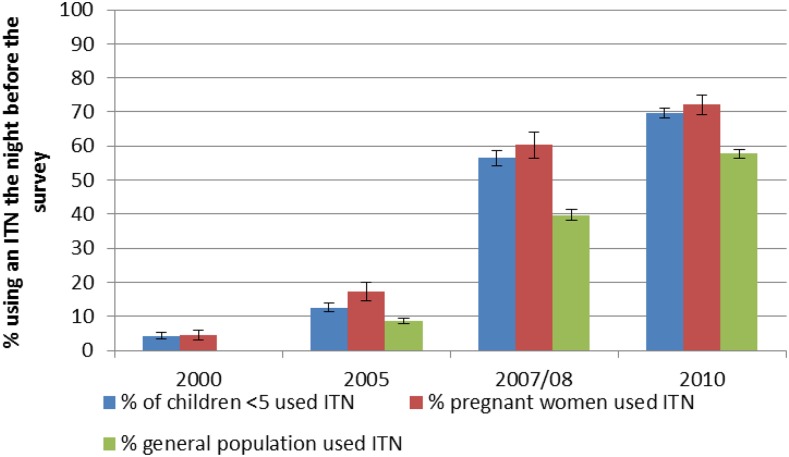
In all households, the proportion of children under five, pregnant women, and all household members who slept under at insecticide-treated net the previous night, 2000–2010. This figure appears in color at www.ajtmh.org.

Ownership of ITNs rose from 15% (95% confidence interval [CI]: 14–16%) in 2005 to 82% (95% CI: 81–83%) in 2010 (Figure 3). Similar to household ownership, use of ITNs increased during 2000–2010 (Figure 4). Although the increase in ITN use was highest in children under 5 years of age (4% [3–5%] in 2000–70% [68–71%] in 2010) and pregnant women (4% [3–6%] in 2000–72% [69–75%] in 2010) increases in ITN use also occurred among the entire population (from 9% in 2005 to 58% in 2010). In households owning at least one ITN, use increased significantly in children under 5 years (from 64% to 75%), and in the general population (from 52% to 67%) comparing data from 2005 to 2010; however, for pregnant women, ITN use (in households owning at least one ITN) did not change significantly, remaining at around 80% throughout the evaluation period.^2^

#### Indoor residual spraying.

Modern IRS with pyrethroid insecticides began late in the evaluation period, in 2007, in three districts in Kigali City and expanded to seven high malaria burden districts in 2009. An estimate of national IRS coverage derived from the 2008 DHS showed that 4% of the population lived in households that had been sprayed. Following the decline in transmission in 2008, the program switched from district-wide coverage to targeted focal spraying of houses in subdistrict areas. As of 2010, approximately 1.3 million people (15% of the population) were protected by IRS. The seven districts sprayed in the 2010 round accounted for more than 70% of the malaria burden in Rwanda.†

#### Case management of fever.

During the evaluation period in Rwanda, case management of fever in young children changed significantly, evolving from presumptive treatment of all fevers with CQ to parasitological testing followed by treatment of positive cases with ACTs, the policies and available tools during this time. Point-of-care options that were initially facility-based have expanded, becoming increasingly available at the community level. According to national survey data, the percentage of children under 5 years of age with recent fever for whom advice or care was sought increased from 26% in 2000 to 48% in 2010. Despite this increase, results suggest that less than half of children with fever were tested for malaria in the same timeframe. Among young children with fever who sought care and who received antimalarials, the percentage who received the recommended first-line treatment of uncomplicated malaria increased significantly from 50% in 2000 to 96% in 2010. At the community level, the proportion of children under five with fever/malaria receiving antimalarials within 24 hours of fever onset increased between 2008 and 2010 (from 62% to 89%). According to the NMCP, the proportion of children receiving parasitological tests for malaria before administration of antimalarials increased from 45% in 2008 to 94% in 2010 as RDTs have been rolled out.

#### Malaria in pregnancy.

Although it is an effective malaria control intervention in other countries, IPTp was only used for a short time in Rwanda. Rwanda continues to implement early detection and treatment of malaria cases and targeted ITN distribution for pregnant women. Use of ITNs by pregnant women increased from 4% (95% CI: 3–6%) in 2000 to 72% (95% CI: 69–75%) in 2010. ANC attendance increased significantly as well, with the percentage of pregnant women attending at least one ANC visit rising from 93% to 98% and the percentage attending at least two visits rising from 79% to 94% over the evaluation period.

### Malaria morbidity data.

#### Health management information system.

**Figure 5.**
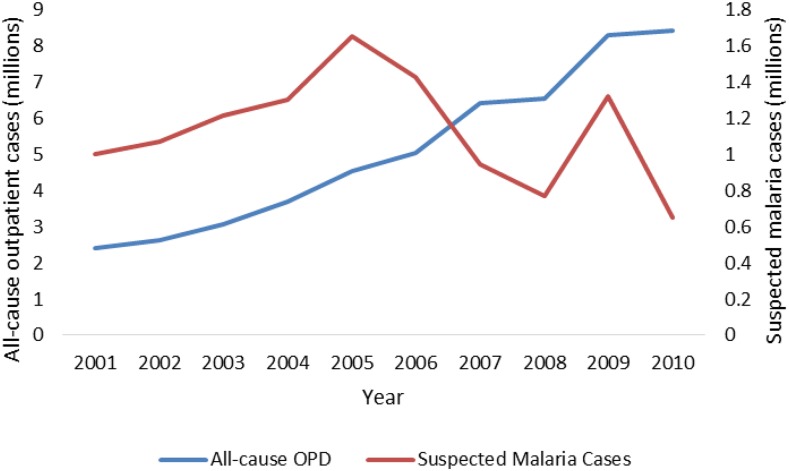
Trend in all-cause out-patient cases and in suspected malaria cases, Health Management Information System 2001–2010. This figure appears in color at www.ajtmh.org.

Trends in reporting and use of outpatient services at health centers (measured by total outpatient department visits) from HMIS data are shown in Figure 5. Between 2001 and 2010 total outpatient visits (for all causes, all ages) increased by 175%, from 2.4 million to 8.5 million. Total (suspected and confirmed) reported malaria cases also increased from 2001 to 2005, but then declined between 2005 and 2008, by 23% over the latter period. In 2009, there was a seasonal increase before returning the lower levels. The total number of malaria cases (suspected and confirmed) reported by health centers increased from 2001 to 2005, but then declined between 2005 and 2010 with a brief spike in cases in 2009. Overall, outpatient (suspected and confirmed) reported malaria cases declined 60% between 2001 and 2010.

**Figure 6.**
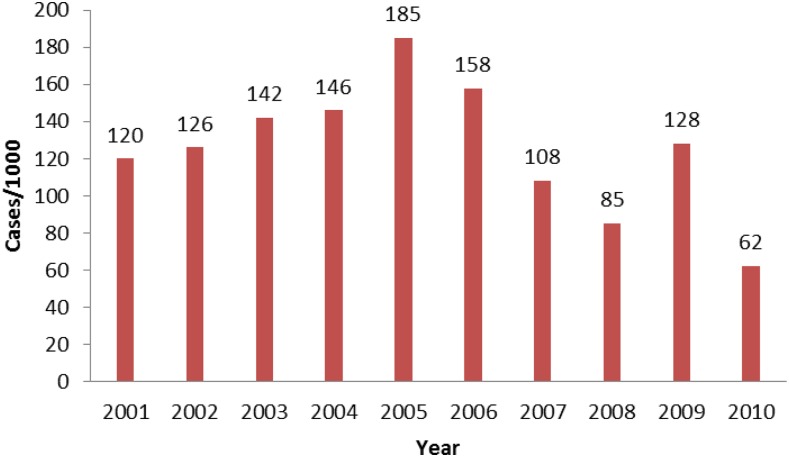
Malaria incidence per 1,000 persons (all age groups), 2001–2010, Health Management Information System. This figure appears in color at www.ajtmh.org.

Malaria incidence is derived from data on new cases and health facility catchment population at risk. Overall, yearly malaria incidence increased from 2001 to 2005 and then declined from 186 cases per 1000 in 2005 to 62 per 1000 in 2010, representing a total decrease of 50% during our evaluation (Figure 6). This reduction occurred simultaneously with significant changes in diagnostic practices and reporting procedures. Parasitological confirmation of suspect cases by the use of microscopy or RDTs increased dramatically during the evaluation period. Diagnostic confirmation was by microscopy only at the beginning of the period, and was done only in facilities that had functional laboratory capacity. RDTs were introduced in 2006, primarily for use by community health workers, whereas health facilities continued to use microscopy for confirmation. By 2009, Rwanda implemented a policy of universal parasitological diagnosis for malaria. The proportion of suspect cases that received laboratory confirmation of malaria increased from 49% in 2009 to 94% in 2010.

TPR is a measure limited to those who used health services (facility- or community-based health worker), received a diagnostic test, and had its result recorded; it was derived from the country’s own routine surveillance. Thus, trends in TPR may reflect actual changes in malaria transmission, provided there was no change in the quality of testing over the evaluation period, and that the susceptibility to malaria was consistent across the population seeking care. Statistical significance of difference in the yearly estimates is dependent on reporting (i.e., numbers of febrile patients who sought care and were tested) which increased over time as access to health services and to diagnostic tests improved. Over the evaluation period in Rwanda, the TPR decreased substantially from more than 50% in 2001 to ∼20% in 2010.

**Figure 7.**
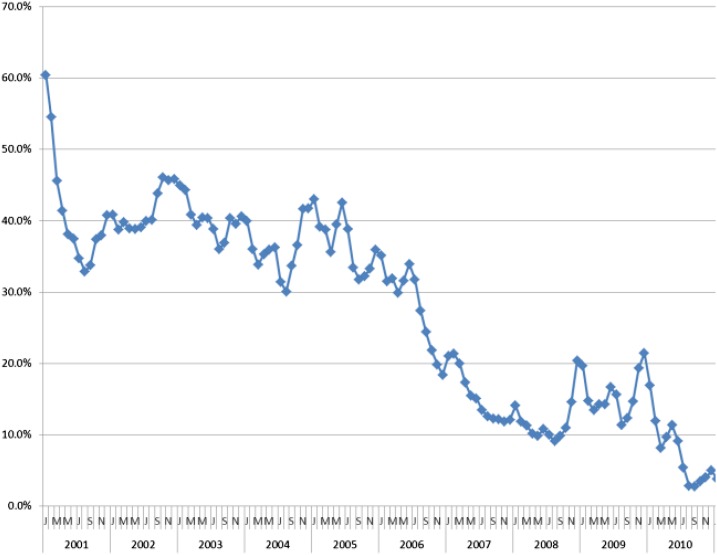
Monthly proportional malaria morbidity in children under five, 2001–2010, Health Management Information System. This figure appears in color at www.ajtmh.org.

HMIS data from health facilities show significant declines in reported outpatient malaria cases, incidence, proportional malaria morbidity, and slide positivity rates from 2000 to 2010. Most of the decreases in these routine morbidity measures accelerated in the second half of the decade (2005–2010) which correlates with scale-up of malaria interventions. Seasonal and annual variations were observed (e.g., increased incidence of malaria in 2009) among the longer term declines observed (Figure 7). These trends in routine data are corroborated by periodic nationally representative household survey data from 2005 to 2010 which show significant decreases in severe anemia and malaria parasitemia among children 6–59 months of age.

#### Malaria-related anemia and parasite prevalence.

National estimates of the prevalence of severe anemia (hemoglobin concentration < 8 g/dL) in children aged 6–59 months are available from the 2005, 2007–2008, and 2010 DHS. Anemia prevalence among children 6–59 months declined significantly from 5.4% (4.6–6.4%) in 2005 to 1.3% (1.0–1.7%) in 2010. The change in prevalence of severe anemia (< 8 g/dL) between 2005 and 2010 differed by location of residence; the indicator decreased from 9% (7–11%) to 2% (1–3%) in the east and 7% (4–13%) to 2% (1–4%) in Kigali City. Prevalence of anemia declined significantly across malaria risk zones, but declines were greatest in the highest risk areas (from 7.7% [6.1–9.7%] to 1.7% [1.2–2.4%] between 2000 and 2010) compared with the others over this period.

**Figure 8.**
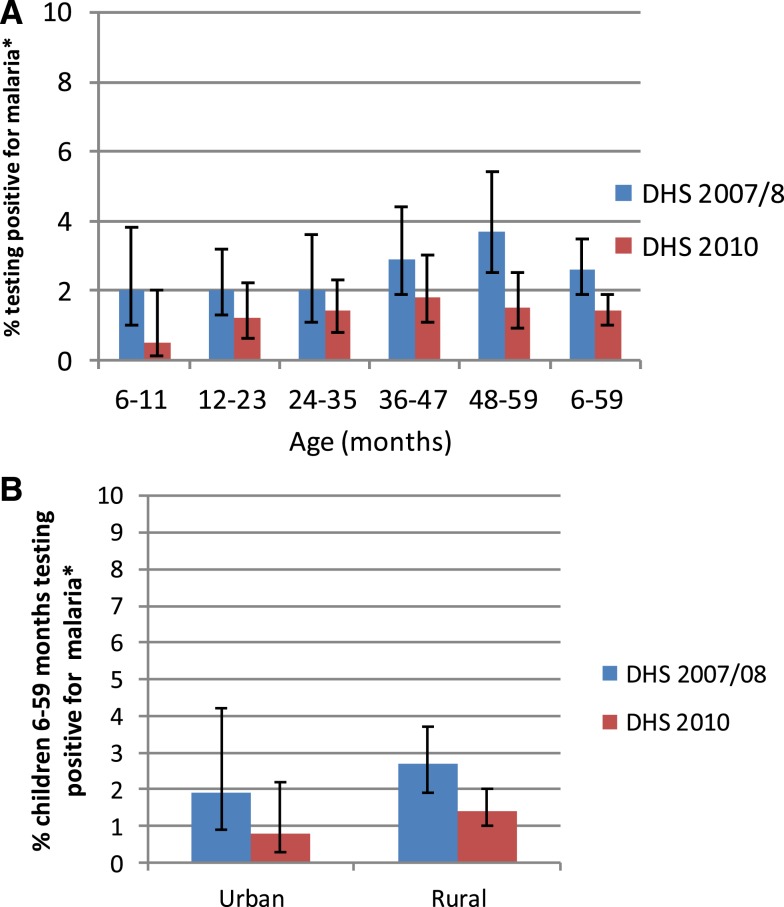
Parasitemia prevalence in children 6–59 months, Rwanda, 2007/2008, 2010, stratified (**A**) by age, (**B**) by household residence, and (**C**) by household wealth. * Measured via microscopy. This figure appears in color at www.ajtmh.org.

The 2007–2008 DHS and 2010 DHS tested children aged 6–59 months for the presence of *Plasmodium falciparum* by RDT and using microscopic examination of thick and thin blood smears. The dates of data collection, December 2007 to March 2008, and September 2010 to April 2011, encompass one of the two annual peak transmission seasons. The parasitemia distribution appears to be very homogeneous across age, urban/rural residence, sex, and wealth strata though this may be due to the low prevalence of malaria parasitemia in Rwanda and the population-based (as opposed to risk based) sampling procedures in the household surveys, (Figure 8 A–C). Parasitemia, as measured by microscopy, decreased significantly between the two surveys in children 6–59 months of age from 2.6% (1.9–3.5%) in 2007–2008 to 1.4% (1.0–1.9%) in 2010; however, no significant differences in parasitemia by age, by household residence, or by household wealth were evident for either survey. In both surveys, parasitemia prevalence was significantly higher in the East than in the North or West Provinces. Regional declines in prevalence over time were not significant.

#### All-cause mortality in children under five.

**Figure 9.**
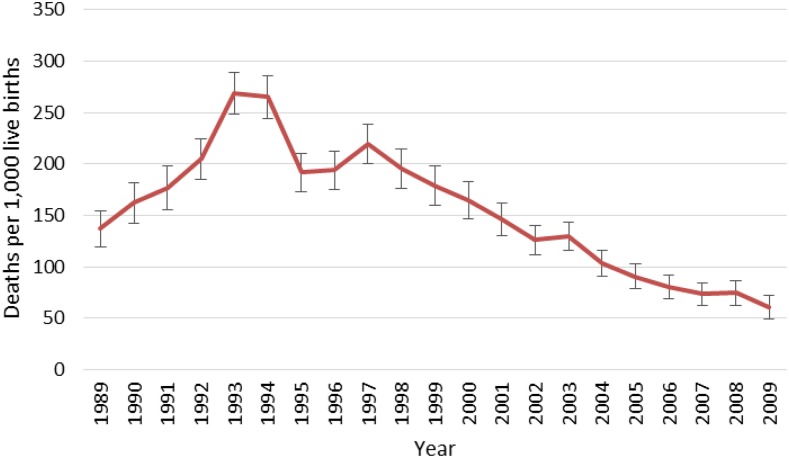
Annual all-cause under-five mortality rates from Demographic and Health Surveys data, 1989–2010. This figure appears in color at www.ajtmh.org.

Estimates of annual ACCM from successive DHS surveys conducted between 1992 and 2010 are presented in Figure 9. All mortality analyses using DHS data represent direct estimates of ACCM for a period of 0–4 years before each survey unless otherwise stated. From 1998 to the end of the evaluation period in 2010, significant reductions in ACCM are evident among all 5-year periods, with more accelerated declines in the later portion of the evaluation period. Between 1996–2000 and 2001–2005 ACCM declined by 22%. Between 2001–2005 and 2006–2010 ACCM declined by 50%. Between 1996–2000 and 2006–2010 ACCM declined by 61%. Observed mortality declines between 1996–2000 and 2006–2010 are lowest in the neonatal and infant age groups (39% and 54% relative reductions, respectively) and are more than 60% for all other age groups. Relative change in mortality increased over time for most age categories; the relative decline in mortality between 1996–2000 and 2001–2005 was less than that for the second half of the decade, 2001–2005 to 2006–2010, Figure 10). Significant reductions in ACCM occurred in all age categories between 1996–2000 and 2006–2010, but the reductions were greatest in children aged 24–59 months at 73%.

**Figure 10.**
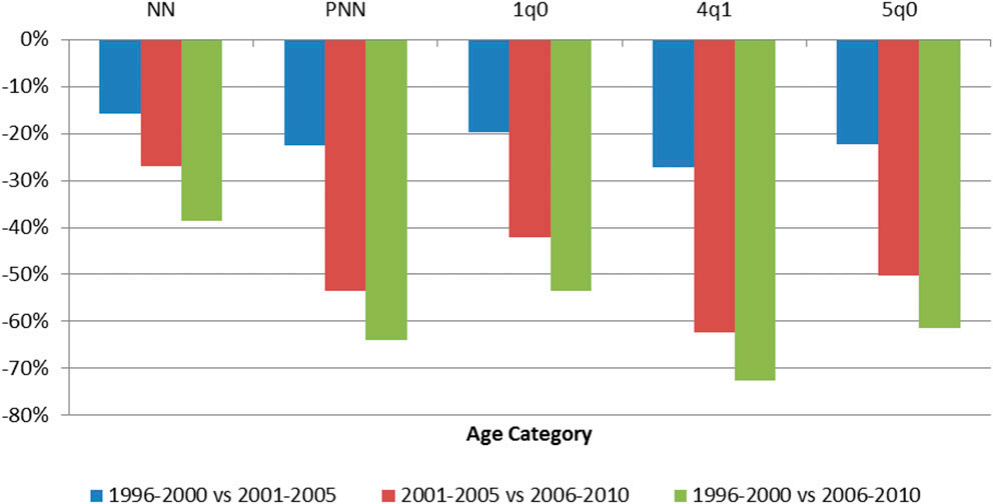
Relative percent change in age-specific childhood mortality in children in Rwanda; a comparison of 5-year estimates from the 1996, 2000, 2005, and 2010 Demographic and Health Surveys. Key: NN = neonatal mortality (first month), per 1,000 live births; PNN = postneonatal mortality (age 1–11 months), per 1,000 live births; _1_q_0_ = infant mortality (first year), per 1,000 live births; _4_q_1_ = child mortality between exact age 1 and exact age 5, per 1,000 children surviving to 12 months of age; _5_q_0_ = under-five mortality, per 1,000 live births. This figure appears in color at www.ajtmh.org.

**Figure 11.**
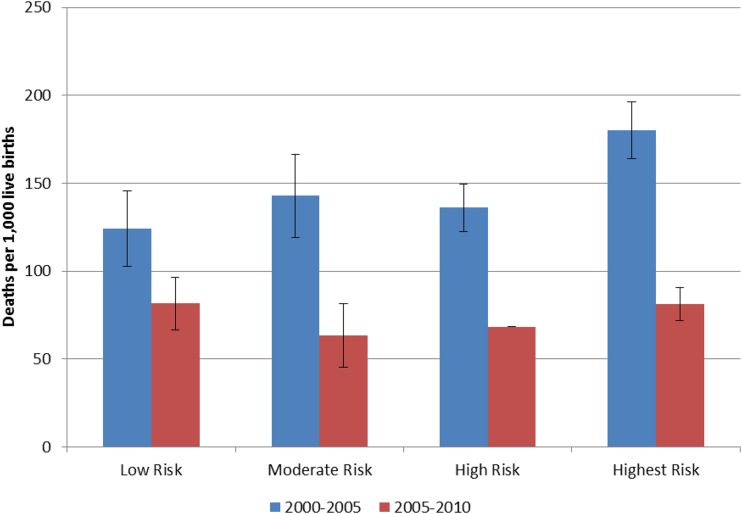
Trends in all-cause child mortality by malaria risk, Rwanda 1996–2000 and 2006–2010. This figure appears in color at www.ajtmh.org.

If a major part of the decline in ACCM was malaria related, we would expect to see a greater decline in mortality, from a higher baseline, among children living in areas of greater malaria risk—as compared with areas of lower malaria risk.^13,14^ We examined trends in child mortality rates stratified by four malaria risk zones. For children under 5 years of age, mortality rates in the four malaria risk categories in the 2000–2005 period were 124, 143, 136, and 180 deaths per 1,000 live births, respectively (Figure 11). Although the mortality rates in the low, moderate, and high risk zones were not significantly different, the highest risk zone had significantly higher mortality than the other three. By 2006–2010, these mortality rates had declined to 82, 63, 68, and 81 deaths per 1,000 live births; there were statistically significant changes in all risk areas. In addition, by the end of the evaluation period, the ACCM in the highest risk zone was not statistically different from the other three zones.

**Figure 12.**
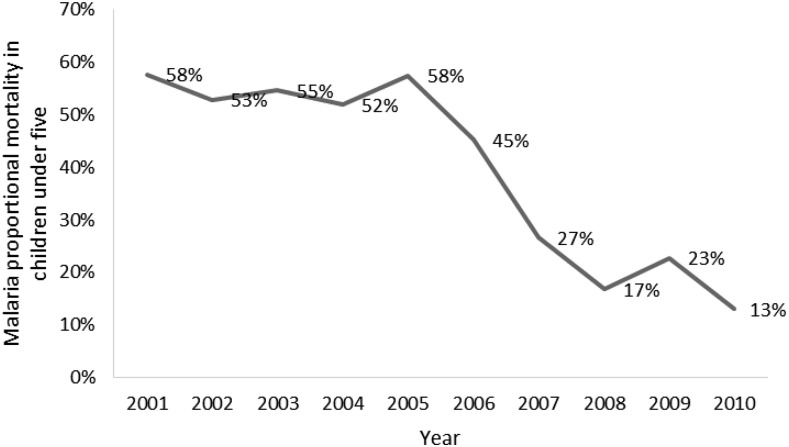
Proportional malaria mortality in children under five, 2001–2010, Health Management Information System. This figure appears in color at www.ajtmh.org.

The data from the national surveillance system was also used to look at the proportion of deaths among children under five which were attributed to malaria. From 2001 to 2005, the proportion stayed relatively flat, but beginning in 2005, the proportion of under five deaths attributed to malaria fell precipitously, from 58% to 13% in 2010 (Figure 12).

#### Contextual determinants of child survival.

Appropriate consideration of contextual factors is essential for ensuring the internal and external validity of impact evaluations of large-scale health programs. Rwanda has experienced many positive developments during the evaluation period, many of which would be expected to lead to improved child survival (summarized in Table 1). Economic poverty, either at the country or individual level, strongly correlates with poorer health outcomes. Gross domestic product (GDP), a standard indicator of population wealth, more than doubled during the intervention period, from $214 per capita in 2000 to $529 in 2010. Similarly, to GDP, women’s literacy is strongly correlated to improved health outcomes for their children. With economic growth, sanitation infrastructure in Rwanda also improved at the community and household levels. Access to a safe source of drinking water and improved toilet facilities increased exponentially over the timeframe. Women also began to stay in school longer and the literacy rate improved from 66% in 2000 [95% CI: 64–68%] to 77% (95% CI: 76–78%) in 2010.

**Table 1:** Changes in contextual factors related to all-cause child mortality in Rwanda, 2000–2010

Survey year Household indicators	2000			2010			% change	Sig.*
	%	95% CI	*n*	%	95% CI	*n*		
Improved water source (protected, borehole, piped), (% households)	40.4	37.1–43.7	9,696	73.8	71.4–76.0	12,540	82.7	S
Improved/not shared pit latrine with slab?	6.0	5.0–7.2	9,696	57.6	56.3–59.0	12,540	860.0	S
Maternal indicators								
Female literacy (%)	66.1	64.4–67.7	10,421	76.9	75.8–78.0	13,671	16.3	S
ANC visits 4+ (% women, most recent live birth, 0–2 years)	10.3	9.2–11.5	5,141	35.4	33.9–37.0	6,405	194.8%	S
Delivery at a health facility (% women, live births in last 0–4 years)	26.5	24.3–28.9	8,188	68.9	67.3–70.5	9,137	132.9%	S
Child health indicators								
Low birth weight < 2500 g (%)	2.2	1.9–2.6	8,188	6.2	5.6–6.9	6,196	181.8	S
Under-fives stunted (%)†	48.3	46.7–50.0	6,514	44.2	42.5–46.0	4,356	−8.5	S
Under-fives underweight (%)†	19.5	18.3–20.7	6,514	11.4	10.4–12.5	4,356	−41.5	S
Under-fives wasted (%)†	8.3	7.5–9.1	6,514	2.8	2.3–3.4	4,356	−66.3	S
DPT3/DPT3-HBV-Hib	86.2	83.7–88.3	1,328	96.8	95.6–97.7	1,616	8.3	S
EPI vaccination coverage	76.0	73.0–78.8	1,330	90.1	88.3–91.7	1,616	12.1	S
Vitamin A supplementation within past 6 months (% children 6–59 months)	68.9	66.9–70.9	6,245	92.9	92.1–93.6	7,873	34.8	S

ANC = antenatal care; EPI = expanded program on immunization; S = statistical significance; WHO = World Health Organization.

* Statistics with non-overlapping 95% confidence intervals are considered significantly different change.

† Definitions and methods per WHO reference population.

In addition to the improvements in general socioeconomic conditions, other favorable changes have occurred in factors directly related to child survival: maternal health indicators improved as more women completed four or more ANC visits (35% [34–37%] in 2010 compared with 10% [9–12%] in 2000) and delivered at a health facility (69% [67–71%] in 2010 versus 27% [24–29%] in 2000). Although the percentage of low birth weight babies increased from 2.2% (1.9–2.6%) in 2000 to 6.2% (5.6–6.9%) in 2010, the absolute numbers remained low. Longer term indicators of nutritional deficiency such as stunting, wasting, and underweight prevalence declined significantly over the evaluation period. Although immunization coverage increased, fairly high coverage of most immunizations already existed at baseline. Similarly, vitamin A coverage was fairly high at the start of the study period with 69% (67–71%) of children under five having received supplementation. This figure increased to 93% (92–94%) by 2010. All of these improvements are likely to positively influence child survival, although the relative importance of each factor is difficult to predict (Table 1).

#### Human immunodeficiency virus and acquired immune deficiency syndrome.

In Rwanda, the first cases of human immunodeficiency virus HIV infection were reported in 1983 and the epidemic rapidly spread. Population trends in prevalence of HIV infection in Rwanda were monitored through the 2005 and 2010 DHS. Among women aged 15–49, HIV prevalence did not change between 2005 and 2010 with an estimated 3.6% infected in 2005 and 3.7% infected in 2010.

Data from the 2010 DHS showed urban/rural disparities in HIV prevalence with the highest burden occurring in Kigali City where 6.7% of men and women aged 15–49 were HIV positive, whereas other provinces had much lower infection rates. Despite these disparities, there has not been a differential change in HIV prevalence by region since 2005. In addition, regional ACCM patterns do not correlate with regional HIV prevalence patterns. This suggests that HIV prevalence over the study period is unlikely to have affected trends in ACCM.

Population-based estimates of HIV infection in children under five are not available. However, analyses based on national models of HIV and acquired immune deficiency syndrome show that the HIV-attributable child mortality per 1000 live births (corrected for other competing causes of mortality) was around 4% in 2000 as compared with 2% in 2010.^25,26^

#### Temperature and rainfall variations.

In Rwanda, malaria transmission is characterized by distinct patterns that seem to follow seasonal rainfall and the spatial distribution of temperature. Rwanda has four seasons: a short rainy season from September to November and a longer season between March and May. Between these seasons are two dry periods, a short one between December and February and a long one from June to August. The mean national annual temperature during 1981–2012 ranged between 18°C and 19°C, but varied according to local topography. The national annual rainfall dated 1981–2013 was approximately 44 inches although there is considerable variation across the country. Throughout the country, minimum and maximum temperatures during the first rainy season have increased over the last three decades, putting cooler areas at increased risk of transmission in the April–June malaria season. There has been significant decline in malaria incidence across Rwanda during 2000–2010 and climate may have aided this decline during significant drought periods. Anomalously high rainfall and temperature appears to be involved in the occasional disruptions in the downward trend of malaria, for example, in 2009. The preintervention period (1996–2000) included the 1997/1998 El Niño, whereas the intervention decade (2001–2010) included two major drought periods which are likely to have aided the control efforts.^9^

#### Decomposition models.

To further explore which malaria control interventions and contextual factors contributed to the declines in ACCM seen across the evaluation period, a decomposition analysis was conducted. The variables examined in the decomposition analysis included demographic variables and measures of socioeconomic status and coverage of a range of maternal and child health interventions, all of which showed statistically significant improvements over the evaluation timeframe during univariate analysis (Table 1). Results show that the distribution of several variables (malaria and nonmalaria related) between surveys explained a significant proportion of the change in ACCM between 2000 and 2010. Important variables included the child’s sex, multiple birth status, ANC, mother’s age at birth, interval between births, and household bed net ownership‡ (Table 2).

**Table 2:** Decomposition model of all-cause child mortality: results comparing Rwanda 2000 and 2010 DHS

Characteristics	Endowments	Coefficients	Model total	Observed total
Child’s sex	0.10*	−5.09		
Multiple birth	0.25***	0.22		
Antenatal care	−11.12***	13.90		
Mother’s age at birth	−1.72***	26.87		
Birth interval	−1.35**	5.53		
Household bednet ownership	−41.71**	−3.07		
Mother used an ITN	−7.80	−0.06		
Mother’s tetanus immunization	−1.03	−6.78		
Household wealth	0.35	1.60		
Mother’s education	−1.09	5.25		
Vaccination coverage	3.33	3.47		
Assisted delivery	−5.62	10.72		
Protected water source	−2.90	20.73		
Improved sanitation	−4.07	−9.15		
Rural residence	−0.31	−47.02		
Constant		−26.23		
Change in ACCM	−74.48***	−9.12	−83.47	−120
% of modeled change	89%	11%	100%	
% of observed change	62%	8%	70%	100%

ACCM = all-cause child mortality; DHS = Demographic and Health Surveys; ITN = insecticide-treated net. Values represent change in numbers of deaths per 1,000 live births between 2000 and 2010 estimated due to changes in variable distributions (endowments) or changes in associations between variables and ACCM (coefficients).

* *P* < 0.05, ** *P* < 0.01, *** *P* < 0.0005.

Decomposition models of child mortality showed that the observed increase in household bed net ownership, from 8% to 94% could have explained as much as 50% of the modeled decline in ACCM between 2000 and 2010 and as much as 35% of the observed decline in ACCM, equivalent to a reduction of 42 deaths per 1,000 live births per year, in the absence of other changes. Improvements in coverage of ANC could have explained an additional decline of 11 deaths per 1,000 live births (13.3% of total modeled ACCM decline and 9.3% of observed ACCM decline). In addition, changes in the distribution of child’s sex, multiple births, mother’s age at birth (18–34 versus < 18 or > 34), and birth intervals (< 24 months) between 2000 and 2010 were all found to contribute significantly to the modeled change in ACCM; however, the modeled changes in ACCM attributable to these variables were small (an increase of 0.1 and 0.25 deaths per 1,000 live births for sex and multiple births, and a decrease of 1.7 and 1.4 deaths per 1,000 live births for mother’s age and birth intervals). Of all of the compositional changes, the increase in household bed net ownership explained the greatest proportion of the observed reduction in ACCM.

## DISCUSSION

Rwanda was very successful at expanding the coverage of malaria-control interventions over the decade of the evaluation period. Bednet coverage increased exponentially such that 82% of households owned an ITN by 2010. IRS, which started later in the decade, covered a large portion of the population in the areas of high transmission by 2010. Similarly, the health sector improved its ability to detect malaria, through widespread use of RDTs, and to treat infections with an effective antimalarial.

The patterns observed in the analysis of the trends in morbidity and mortality declines are consistent with the hypothesis that malaria control interventions had an impact on reducing transmission. In terms of morbidity markers, the prevalence of severe anemia (< 8 g/dL) declined significantly across the decade, with the greatest declines seen in children 6–29 months, the age group in which anemia is most commonly associated with malaria, and in the geographic regions at highest risk for malaria. Data from the health facilities show evidence of significant declines in outpatient malaria cases as well as declines in malaria incidence, proportional malaria morbidity, and TPRs from 2000 to 2010. Most of the decreases in these morbidity measures occurred in the second half of the decade, from 2005 to 2010, subsequent to the introduction and roll out of ITNs in 2006, the introduction of IRS in 2007, and the introduction of ACTs and home-based management of malaria in 2006. These findings are consistent with other published analyses of Rwanda’s surveillance data.^27^ Although malaria morbidity was declining throughout the decade, the clear temporal association with the rapid expansion of malaria control interventions further enhances the plausibility argument that the interventions had a substantial effect on reducing malaria burden.

As seen in many other countries in sub-Saharan Africa, a significant decline in ACCM occurred in Rwanda between 2000 and 2010, a period of intense investment in malaria control interventions. The United Nations reported that 48 million children’s lives were saved between 2000 and 2015 by reductions in under-five mortality, with a 3.9% annual reduction in ACCM.^25^ In sub-Saharan Africa the annual reduction was 4.1% and in Rwanda 5.2%. According to DHS data, ACCM in children in Rwanda decreased by 61% percent between 2000 and 2010; from 196 to 76 deaths per 1,000 live births. This decline may be attributable to a number of child survival interventions, including increases in coverage of malaria control interventions. To further examine this decline, mortality in children under five was stratified by residence (i.e., urban or rural), age (e.g., 6–23 months, 24–59 months), and underlying malaria risk. The mortality declines were larger in children residing in rural areas (62%) as compared with children living in urban areas (53%). During the same period, the relative decline in mortality in children 6–23 months (69%) who are at higher risk of severe malarial anemia and mortality was similar to the relative decline in children 24–59 months (73%). Mortality declines from 2000 to 2010 were also larger in regions with moderate to highest malaria risk (50–56% reductions) than in those with lower malaria risk (34%). As with the indicators of morbidity, the most significant declines in ACCM over the period 2000–2010 occurred in the latter part of the decade (2006–2010), corresponding to the period when malaria control interventions began to have an impact nationwide.

The declines in morbidity and mortality seen in this analysis must be interpreted in the context of other contextual factors which may have influenced these declines. Key among these factors is the substantial economic growth seen during the evaluation period. Economic growth leads to improvements in sanitation, housing, education, and other factors that can have indirect impacts on health outcomes. This economic growth likely had a strong indirect influence on health outcomes, particularly mortality, though it is important to note that declines in the mortality rate began almost a decade before the GDP experienced a substantial increase.

Other interventions targeting child health improved during the evaluation period as well. ANC use increased, as did safe delivery services, though the magnitude of uptake of these maternal health interventions was not nearly as substantial as the uptake of malaria control. Vitamin A supplementation increased, as did vaccination coverage. However, these interventions were already reaching 70–75% of the under-five population at baseline. The proportional increase in these interventions is less likely to have influenced the mortality figures as compared with the much greater increase in malaria control interventions.

Climate is an important driver of malaria transmission in Rwanda where the rainy season is bimodal and the varied topography impacts local temperatures. Although there has been significant decline in malaria incidence across Rwanda since 2000, climate is likely involved in the occasional disruptions in the downward trend of malaria, most likely due to abnormal rainfall and temperature.

Decomposition model results support the plausibility argument, showing the relative importance of increasing bednet ownership compared with other interventions on declines in ACCM during the evaluation period. Decomposition models show that the observed increase in household bed net ownership from 8% to 94% could explain as much as 50% of the observed decline in ACCM between 2000 and 2010. Although this estimate is much greater than the 17% protective efficacy from earlier cluster-randomized trials,^27^ it is similar to results from other, more recent analyses. Lim and others showed that household ownership of ITNs was associated with a 55% decrease in risk of ACCM (95% CI: 28–72%) among children 1–59 months of age using data from the Rwanda 2007–2008 Interim DHS.^28^ Across 29 surveys, household ownership of at least one ITN was associated with a 23% reduction in under-five deaths (95% CI: 13–31%).

### Limitations.

There are several limitations inherent in the data and methods used in this study. The primary outcome of interest, ACCM, is derived from cross-sectional surveys and represents the midpoint of a period of 5 years before the survey date. Thus, the mortality estimates are not directly linked temporally with the interventions. ACCM, while a robust and standardized indicator, is not specific to malaria. Indeed, as prevalence of malaria declines, as we see in Rwanda, the portion of ACCM due to malaria diminishes to a point where ACCM declines are no longer a valid marker of the success of malaria control programs. The use of a child mortality indicator as an end point also does not reflect the impact of the malaria control program on older children and adults. These issues are discussed in greater detail in Ye and others in this supplement.

Other variables taken from the cross-sectional surveys are subject to the biases inherent in this type of data source. The data are self-reported, and for some variables there is a recall period which can create further bias in the data. For indicators on ITN use, reported use the night before the survey (the variable used in the study) does not necessarily indicate consistent use throughout the high transmission period each year and does not reflect the duration of time prior to death that a child may have used an ITN.

Data from the routine information system also presents some limitations which must be considered. Over the 10-year period of this study, the public health system of Rwanda saw sizeable growth and improvement. New health centers were constructed across the country which increased access to services. Changes in diagnostic and treatment practices, as well as care-seeking behaviors (discussed earlier), over the timeframe mean that trends in case data from facilities are difficult to interpret. In addition, the study team did not have access to the raw HMIS data, but instead had to rely on aggregate data from reports produced by the Ministry of Health. This limited the analysis that could be conducted on the surveillance data and it is used here for illustrative and supplemental purposes.

## CONCLUSION

Rwanda made dramatic progress in increasing population coverage of malaria prevention and treatment measures in the decade from 2000 to 2010. In this timeframe, Rwanda saw a substantial increase in bednet ownership, use among children under five, and sizeable gains in access to diagnosis and treatment of malaria. Over the same period, ACCM declined 61% and prevalence of severe anemia in children 6–23 months declined 71%. Larger declines in child mortality and severe anemia were observed in rural areas, where the burden of malaria is higher compared with urban areas. Multivariable models of the change in child mortality between the 2000 and 2010 DHS reveal the importance of increasing bed net ownership in explaining the observed mortality declines. Taken as a whole, the evidence supports the conclusion that malaria control interventions contributed to the observed decline in child mortality in Rwanda from 2000 to 2010, even in a context of improving socioeconomic, maternal, and child health conditions.

## Footnotes

† It should be noted that the targeted spraying in these seven districts only focused on the highest burden communities, not every household in the district.

‡ ITN ownership was included as a proxy for use, as the survey datasets do not include information on net use for children who died.
